# Deep Reinforcement Learning Approach with Multiple Experience Pools for UAV’s Autonomous Motion Planning in Complex Unknown Environments

**DOI:** 10.3390/s20071890

**Published:** 2020-03-29

**Authors:** Zijian Hu, Kaifang Wan, Xiaoguang Gao, Yiwei Zhai, Qianglong Wang

**Affiliations:** School of Electronic and Information, Northwestern Polytechnical University, Xi’an 710129, China; huzijian@mail.nwpu.edu.cn (Z.H.); cxg2012@nwpu.edu.cn (X.G.); zyw@mail.nwpu.edu.cn (Y.Z.); wql1995@mail.nwpu.edu.cn (Q.W.)

**Keywords:** UAV, motion planning, deep reinforcement learning, multiple experience pools

## Abstract

Autonomous motion planning (AMP) of unmanned aerial vehicles (UAVs) is aimed at enabling a UAV to safely fly to the target without human intervention. Recently, several emerging deep reinforcement learning (DRL) methods have been employed to address the AMP problem in some simplified environments, and these methods have yielded good results. This paper proposes a multiple experience pools (MEPs) framework leveraging human expert experiences for DRL to speed up the learning process. Based on the deep deterministic policy gradient (DDPG) algorithm, a MEP–DDPG algorithm was designed using model predictive control and simulated annealing to generate expert experiences. On applying this algorithm to a complex unknown simulation environment constructed based on the parameters of the real UAV, the training experiment results showed that the novel DRL algorithm resulted in a performance improvement exceeding 20% as compared with the state-of-the-art DDPG. The results of the experimental testing indicate that UAVs trained using MEP–DDPG can stably complete a variety of tasks in complex, unknown environments.

## 1. Introduction

The number of applications for unmanned aerial vehicles (UAVs) is widely increasing in the civil arena such as surveillance [1,2], delivery of goods [3,4], power line inspection [5,6], and mapping [7,8]. In the majority of these applications, it is necessary for UAVs to plan their motion such that they can perform their tasks while avoiding threats in complex, unknown environments.

Many traditional path planning algorithms, such as A* algorithm, visibility graph algorithm, and free space algorithm, are used to solve the motion planning problem of UAV, but these methods can usually only achieve good results when the environment or map is known. Under real circumstances, the task environment of UAV is often unknown or not completely known. Simultaneous localization and mapping (SLAM) maps the unknown environment according to position and sensor information of the UAV during its movement in the environment so as to implement the automatic motion planning of the UAV according to the drawn map. Simultaneous localization and mapping make up for the limitations of traditional path planning methods in unknown environments, but these kinds of methods also have a large disadvantage: it is difficult to solve dynamic environment problems. Once the environment changes, the map needs to be redrawn which will seriously affect the mission efficiency of the UAV.

With the rise of artificial intelligence, reinforcement learning (RL) has become an effective method to solve the above problems. Through continuous interaction between the UAV and the environment, the UAV trained by RL can efficiently complete tasks in complex environments. With the increasing complexity of the environment, deep reinforcement learning (DRL) with neural networks can be applied to environments that are closer to reality. Many studies used DRL to solve the autonomous motion planning (AMP) problem of UAV and achieved good results, but these studies still have some shortcomings: (1) the models of the UAV and environment can be more complex and realistic; (2) the convergence speed and convergence results of the algorithm can be improved.

To address these problems, explorations and experiments were conducted in this study. The main contributions of this study are as follows:

1. Construction of a complex 3D simulation environment for AMP.

A universal environment based on the parameters of the real UAV was built. The UAV, obstacles, etc., in this environment were decided in equal proportion according to actual situations. The complete kinematics model of the UAV enables the environment to better simulate real situations. By changing the environment, simulations of many different tasks can be implemented to test the performance of the algorithm.

2. A multiple experience pools DRL (MEP-DRL) framework was designed to solve the AMP problem in complex, unknown environments.

“Experience” usually refers to a set of data containing state, action, reward, and other information in RL. The agent optimizes its action strategy through continuous learning of experiences. The quality of the experiences determines the effectiveness of the training. Therefore, in order to improve the quality of the experiences learned by the agent, multiple experience pools are used to store different kinds of experiences in this paper. In the learning process, dynamically adjusting the proportion of the learning experiences obtained from each experience pool not only solves the AMP problem but also effectively improves the convergence efficiency and results.

3. A method combining model predictive control (MPC) and simulated annealing (SA) to generate expert experiences was introduced.

The AMP problem is a very complicated decision-making problem. In real situations, expert experiences can greatly improve UAV control. An MPC-SA architecture is designed, in which the MPC predicts the future, and then the SA selects the best outcome from the predicted results, thereby achieving a good simulation of expert experiences.

The remainder of this paper is structured as follows: The related work is discussed in Section 2. The AMP problem of a UAV is formulated in Section 3. Section 4 presents the proposed MEP–DDPG algorithm. The simulation environment is introduced in Section 5. The training and testing experiment results are presented and discussed in Section 6. Finally, Section 7 concludes this paper and envisages some future work.

## 2. Related Work

A multitude of methods ranging from offline to online methods have been proposed to address UAVs’ AMP problems. Offline-based methods do not learn during the planning process and maintain fixed rules or strategies from beginning to end. Many well-known path planning algorithms fall into this category, the most typical of which is the A* algorithm. Yang [9] described a UAV fast path planning method that uses an improved sparse A* algorithm to significantly improve the search efficiency. Wang [10] proposed using an enhanced sparse A* search method based on Dubins path for UAV path planning that can determine the optimal path with less planning time. Ren [11] proposed a three-dimensional (3D) UAV path planning algorithm based on using an A* algorithm to solve the path planning problem in a complex environment. These methods have achieved good results in specific environments, but they do not perform well when planning in unknown environments. In comparison, simultaneous localization and mapping (SLAM), which is one of the online-based methods, can successfully solve the AMP problem even when the environment is unknown [12,13,14,15]. Wang [12] presented a complete navigation system for an indoor UAV that was based on SLAM, and this system was able to ensure that the UAV could fly along the internal walls of a room without collisions. Cui [13] combined SLAM with an online path planning module to allow UAVs to navigate autonomously through a foliage environment. However, since SLAM needs to model the environment online first, if the environment changes, it could perform poorly. In addition, the efficiency of such methods depends on the complexity of the environment. The more complex the environment, the more time it takes to map.

To overcome the shortcomings of the methods described above, other studies [16,17,18,19] have proposed using reinforcement learning (RL), an online learning method, for UAV motion planning. Reinforcement learning can solve the UAV’s AMP problem with navigating in an unknown environment, but as the environment becomes more complex and closer to reality, the “dimension curse” problem limits its development. 

In 2013, DeepMind innovatively combined deep learning (DL) with RL to form a new hotspot in the field of artificial intelligence which is known as DRL [20]. By leveraging the decision-making capabilities of RL and the perceived capabilities of DL, DRL has been proven to be efficient at controlling UAV [21,22,23,24,25,26,27,28,29,30,31]. Zhu [21] proposed a framework for target driven visual navigation, this framework addressed some of the limitations that prevent DRL algorithms from being applied to realistic settings. Zhang [22] presented a coarse-to-fine approach with RL to address the aspect ratio variation issue for target tracking in UAV. Bøhn Eivind [23] proposed a DRL controller to handle the nonlinear attitude control problem, enabling extended flight envelopes for fixed-wing UAVs. Tai [24] designed a mapless motion planner with DRL which can navigate the nonholonomic mobile robot to the desired targets without colliding with any obstacles. Pfeiffer Mark [25] presented and analyzed an approach that combines the advantages of both imitation learning and DRL for target-driven map-less navigation. Han [26] introduced a double deep Q-Network (Double DQN) [32] that utilized a priority sample replay method, and this demonstrated better results than DQN [20] and Double DQN when UAVs navigated through a 3D obstacle avoidance environment. Kersandt [27] used DQN, Double DQN, and Dueling DQN [33] in the same UAV control mission and compared each of these methods. Singla [28] designed a deep recurrent Q-Network [34] with temporal attention that exhibited significant improvements over DQN and D3QN [32] for UAV motion planning in a cluttered and unseen environment. For the autonomous landing task of UAV, Polvara R [29] introduced a sequential DQN which is comparable with DQN and human pilots while being quantitatively better in noisy conditions. Wang [30] proposed a fast recurrent deterministic policy gradient algorithm to address the UAV’s autonomous navigation problem in a large-scale complex environment. Rodriguez-Ramos Alejandro [31] addressed the continuous UAV landing maneuver on a moving platform by means of the DDPG algorithm, trained in simulation, and tested in real flights. 

These studies demonstrate that DRL has already successfully addressed several AMP problems, but as the unknown environment becomes increasing complexity, there are still some challenges that must be overcome:

1. The models of the UAV and environment are simplified.

Previous studies have focused on UAV navigation. Therefore, the UAV model has been simplified. For example, in real situations, the motion space of a UAV is continuous, whereas in some studies [26,27,28], the motion space of a UAV is considered discrete. Furthermore, the flight speed of a UAV is variable within a certain range, where Reference [30] regards UAV speed as a fixed value. In addition to these issues, the environment should also be as realistic as possible. Otherwise, these simplifications of the UAV model will make the learning process simpler; however, they may lead to poor practicability.

2. The convergence speed and results can be improved.

It is well known that DRL, which is the approach based on artificial neural networks, involves many hyperparameters, and its performance is often inextricably linked to the setting of these hyperparameters. In addition, other factors such as different algorithm frameworks and reward functions can also have a significant impact on the results. Therefore, it is essential to use appropriate strategies to optimize these factors and improve the convergence speed as well as the results.

The above problems are discussed and solved in detail in the following sections of this study.

## 3. Problem Formulation

### 3.1. UAV‘s AMP

**Figure 1:**
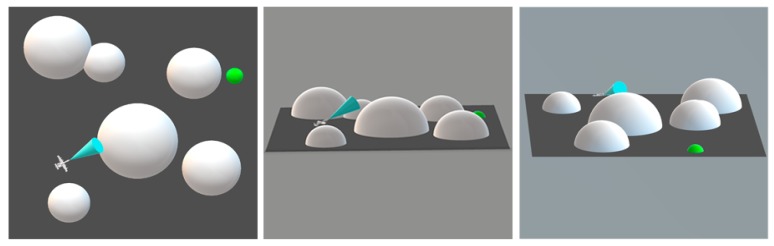
Different perspectives of an AMP problem. **Left**: top view; **Middle and Right**: side views from different perspectives.

As mentioned earlier, in actual applications of UAVs, AMP is an essential technology that determines if the UAV can successfully complete the task. If the UAV cannot appropriately control its own posture and movements, it may result in a failure of the mission and may also cause damage to surrounding objects. For example, when a UAV is delivering goods, it may cause injury to people or vehicles if it is flying too low or too fast. Figure 1 is a schematic diagram of an AMP problem from different perspectives.

The blue cone in the figure represents the range that the UAV can sense through the sensor. The white hemispheres represent dangerous areas such as buildings in ground search tasks or an adversary’s radar monitoring range. The green hemisphere represents the final destination that the UAV needs to reach. In short, the AMP problem is how to make the UAV automatically use the limited information obtained inside the range of the blue cone to adjust its posture and reach the position of the green hemisphere efficiently without entering the white hemispheres areas.

#### 3.1.1. Motion Planning Framework for UAVs

In the real world, UAV motion planning can be divided into three modules: navigation, guidance, and control.

**Figure 2:**
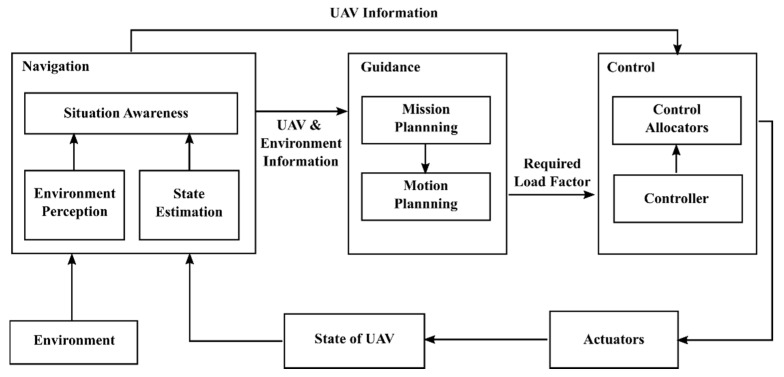
Unmanned aerial vehicle (UAV) motion planning framework for real-world applications.

As shown in Figure 2, the three modules of navigation, guidance, and control complete the motion planning for UAV. The navigation module is primarily responsible for sensing which includes perceiving environment and estimating the state of the UAV and passing the perceived information to other modules. The perceived information usually includes the UAV’s position, speed, flying height, wind speed, target position, etc. The guidance module plays a planning role throughout the closed loop. It analyzes the information obtained about the UAV and environment, plans the motion of the UAV in combination with the scheduled flight mission, and outputs the required load factor to the other modules. When the control module receives the required load factor, the required load factor will be converted into different actuator commands and then passed to the corresponding actuators. These actuators, such as engines and rudders, can execute the corresponding commands and achieve stable control of the UAV. 

**Figure 3:**
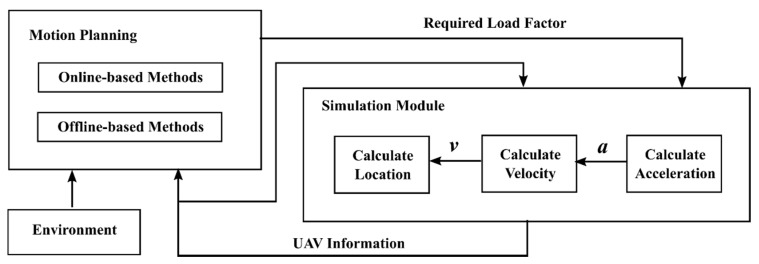
UAV motion planning framework in the simulation environment.

In the simulation environment, the navigation, guidance, and control modules do not operate independently. As shown in Figure 3, the motion planning module directly replaces the navigation and guidance modules. In addition, the control module and the UAV model are integrated into the simulation module. The main work of the motion planning module is integrating the current information of environment and the UAV and using an offline or the online-based method to plan the motion of the UAV according to the UAV’s mission. In addition, based on the action to be performed by the UAV, the motion planning module will calculate the required load factor of this action and transfer it to the simulation module. The simulation module calculates the UAV’s acceleration, velocity, and position based on this load factor and then adjusts the attitude of the UAV model and displays it on the simulation interactive interface.

#### 3.1.2. UAV Model

Previous studies focused more on the motion planning module and simplified the simulation module, while the UAV model used in this study was as realistic as possible. Here, this paper assumed that the velocity direction of the UAV’s velocity was consistent with the direction of the UAV’s axis. When the motion planning module outputs the required load factor $n_{u}$ at the current moment, according to the reference [35], the acceleration $a_{u}$ at the current moment can be calculated as follows:

(1) $$a_{u}=\left[\begin{array}{c} a_{ux}\\ a_{uy}\\ a_{uz} \end{array}\right]=n_{u}\cdot g=\left[\begin{array}{c} n_{ux}\\ n_{uy}\\ n_{uz} \end{array}\right]g$$

Defining Δt as the simulation step, the velocity $v_{u}$ at the current moment can be obtained from the velocity $v_{0}$ at the previous moment:

(2) $$v_{u}=v_{0}+a_{u}\cdot \Delta t=\left[\begin{array}{c} v_{0x}+a_{ux}\cdot \Delta t\\ v_{0y}+a_{uy}\cdot \Delta t\\ v_{0z}+a_{uz}\cdot \Delta t \end{array}\right]$$

Similarly, the current UAV position $p_{u}$ can be calculated from the position $p_{0}$ at the previous moment:

(3) $$p_{u}=\left[\begin{array}{c} p_{ux}\\ p_{uy}\\ p_{uz} \end{array}\right]=p_{0}+v_{u}\cdot \Delta t=\left[\begin{array}{c} p_{0x}+v_{ux}\cdot \Delta t\\ p_{0y}+v_{uy}\cdot \Delta t\\ p_{0z}+v_{uz}\cdot \Delta t \end{array}\right]$$

**Figure 4:**
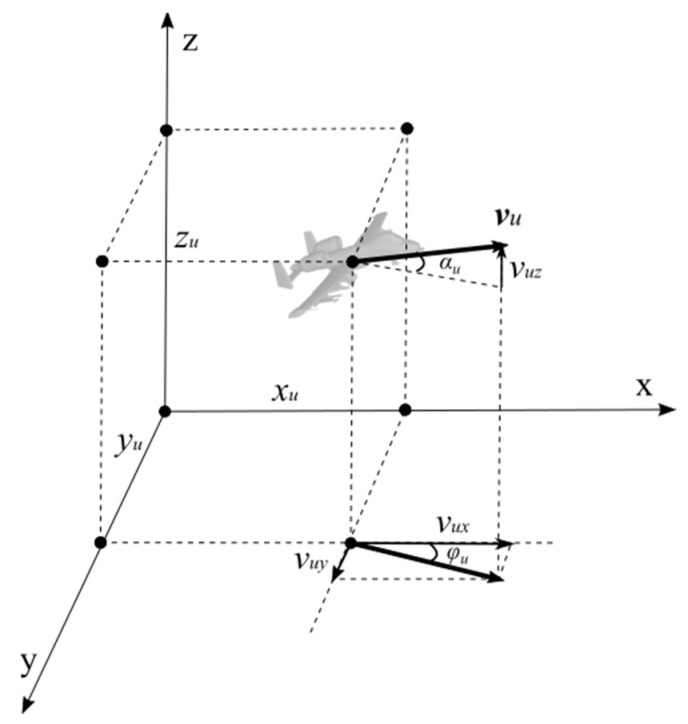
UAV attitude angles calculated in a 3D environment.

At the same time, the yaw angle $\varphi_{u}$ and pitch angle $\alpha_{u}$ of the UAV at the current moment can be calculated according to the direction of the velocity (Figure 4):

(4) $$\varphi_{u}=atan\left(\frac{v_{uy}}{v_{ux}}\right)$$

(5) $$\alpha_{u}=atan\left(\frac{v_{uz}}{\sqrt{v_{ux}{}^{2}+v_{uy}{}^{2}}}\right)$$

So far, all the required UAV information has been obtained. In this paper, the vector $\xi_{u}={\left[p_{ux},p_{uy},p_{uz},\varphi_{u},\alpha_{u},∥v_{u}∥\right]}^{T}$ represents the UAV’s state for calculation and description.

### 3.2. RL for UAV’s AMP

The UAV’s AMP problem is a typical agent decision-making problem [26], so RL-based approaches can be used to address it. In 1957, Bellman [36] proposed a model for sequential decisions known as the Markov decision process (MDP). Almost all RL problems can be modeled as an MDP and shown as a 5 tuple (S,A,P,R,γ), where S is the state space, which is the set of all states that an agent can reach in a specific environment; A represents the action space in the environment; P is the probability of executing an action a from state s to state $s^{\prime }$; R represents the reward received by the agent after executing action a and transiting from state s to state $s^{\prime }$ (where a∈A and $s,s^{\prime }\in S$); γ∈[0,1] is the discount factor which determines the importance of current or future rewards [37].

The interaction process between the UAV and the environment can be constructed using MDP: At each discrete time step t, the state of the UAV is $s_{t}$, and the action $a_{t}$ is performed based on this state. After this time step, the UAV receives the reward $r_{t}$ from the environment and reaches the next state $s_{t+1}$. The action selected by the UAV at each time step is determined by π, which is the collection of policies. For example, an element π(a|s) from π represents the probability that the UAV takes an action a at a certain state s. The goal of RL is to find the optimal policy $\pi^{*}$, and to obtain the largest cumulative reward $R_{t}$ if policy $\pi^{*}$ is followed:

(6) $$R_{t}=\overset{T}{\underset{t\prime =t}{\sum }}\gamma^{t\prime -t}r\left(s_{t\prime },a_{t\prime }\right)$$

where T represents the end time step of an episode.

The state-action value function $Q^{\pi }\left(s,a\right)$ represents the expected episode cumulative reward obtained by the UAV when performing the action a in accordance with the policy π in the current state s:

(7) $$Q^{\pi }\left(s,a\right)=E\left[R_{t}|s_{t}=s,a_{t}=a,\pi \right]$$

When the optimal policy $\pi^{*}$ is followed:

(8) $$Q^{*}\left(s,a\right)=\underset{\pi }{max}E\left[R_{t}|s_{t}=s,a_{t}=a,\pi \right]$$

From the Bellman equation [36]:

(9) $$Q^{*}\left(s,a\right)=E\left[r_{t+1}+\gamma \underset{a_{t+1}}{max}Q^{\pi }\left(s_{t+1},a_{t+1}\right)|s_{t}=s,a_{t}=a\right]$$

Constantly iterating Formula (9) will eventually converge the state-action value function to obtain the optimal policy [38]:

(10) $$\pi^{*}=\underset{a\in A}{argmax}Q^{*}\left(s,a\right)$$

The state-action value function can only be used to address the discrete action space problem, because in continuous spaces finding the greedy policy requires an optimization of $a_{t}$ at every timestep; this optimization is too slow to be practical with large, unconstrained function approximators and nontrivial [39]. While in the real world, the action space of a UAV is continuous, which requires the addition of some policy-based RL methods. These are explained in Section 4.

## 4. MEP–DDPG for Motion Planning

### 4.1. DDPG

RL methods are mainly classified into two types of value-based methods and policy-based methods. Value-based methods output the state-action values and select the action with the highest value. Common methods in this type include Q-learning [40] and DQN. Policy-based methods directly output the probability of the next action, and select the action based on the probability. This type of method is suitable for non-continuous and continuous motion problems. A typical method of the policy-based methods is policy gradient [41].

Lillicrap [39] used the idea of extending DQN based on Q-learning to transform the deterministic policy gradient (DPG) [42] and proposed a DDPG algorithm based on the actor–critic framework. In the actor–critic framework, the actor network chooses action based on probability. The critic network evaluates the action selected by the actor network. The actor network then modifies the probability of action selection based on the evaluation of the critic network. Because of this framework, DDPG could handle problems with both high-dimensional continuous state space and high-dimensional continuous action space [43]. In DDPG, the double-network structure cuts off the correlation and improves the learning efficiency of the neural networks. Consequently, there are target-net and eval-net in both the actor network and the critic network.

The critic network updates the network parameters by minimizing the loss function $L\left(\theta^{Q}\right)$:

(11) $$\{\begin{array}{c} L\left(\theta^{Q}\right)=\frac{1}{N}\sum_{i}{\left(y_{i}-Q\left(s_{i},a_{i}|\theta^{Q}\right)\right)}^{2}\\ y_{i}=r\left(s_{i},a_{i}\right)+\gamma Q^{\prime }\left(s_{i+1},\mu^{\prime }\left(s_{i+1}|\theta^{\mu^{\prime }}\right)|\theta^{Q^{\prime }}\right) \end{array}$$

where $\theta^{Q}$ and $\theta^{Q^{\prime }}$ represent the parameters of eval-net and target-net in the critic network respectively, $\theta^{\mu^{\prime }}$ means the parameters of target-net in actor network, N is the number of experiences used to learn.

The actor network uses the Monte Carlo sampling method to approximate the expected value and approximates its network parameters using the chain rule:

(12) $$\nabla_{\theta^{\mu }}J\approx \frac{1}{N}\underset{i}{\sum }\nabla_{a}Q\left(s,a|\theta^{Q}\right){|}_{s=s_{i},a=\mu \left(s_{i}\right)}\nabla_{\theta^{\mu }}\mu \left(s|\theta^{\mu }\right){|}_{s_{i}}$$

where $\theta^{\mu }$ represents the parameters of the eval-net actor network.

During the training process, only the eval-net parameters are required, and the parameters of the target-net are soft-updated by eval-net at regular intervals:

(13) $$\theta^{\prime }\leftarrow \tau \theta +\left(1-\tau \right)\theta^{\prime }$$

where the range of τ is [0,1].

This soft-updated method makes the problem more similar to supervised learning, which improves its stability.

The DDPG also uses the experiences playback mechanism [20] to store the experience $\left(s_{t},a_{t},r_{t},s_{t+1}\right)$ generated by the interaction between the actor network and the environment in the experience pool. During network training, samples are randomly selected from the experience pool each time there is a network parameter update. However, the experiences in the experience pool are not all useful for learning. In particular, during the early stages of training, a majority of the samples in the experience pool are not useful. Improving the quality of each sampling experience will have a significant impact on the efficiency of the DDPG algorithm.

### 4.2. MEP–DDPG Framework

As mentioned in Section 3, UAV motion planning represents a complicated problem. In practice, UAVs are often controlled by operators on the ground using remote control equipment. When using RL to realize UAV’s AMP, if we can add human expert experiences to the learning process, the UAV may learn better strategies by combining expert experiences with the experiences it has explored. An MEP–DDPG framework is designed to incorporate expert experiences into the DRL process.

**Figure 5:**
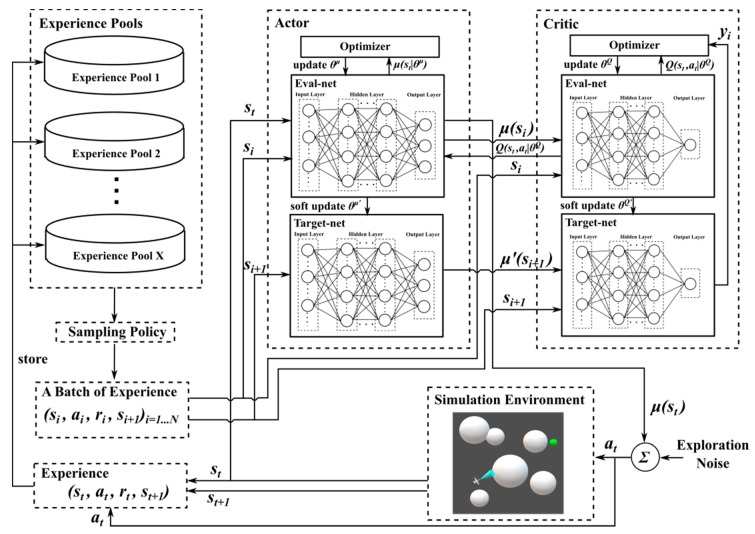
Framework of MEP–DDPG (experience pools are used to store different kinds of experiences; the actor network is responsible for selecting actions; the critic network is responsible for evaluating selected actions).

As shown in Figure 5, multiple experience pools included in MEP–DDPG are used to store different types of experiences. At each time step t, the UAV performs the action $a_{t}$, and through interaction with the environment, an experience $\left(s_{t},a_{t},r_{t},s_{t+1}\right)$ is generated. According to the method of generating this action (whether generated under the guidance of experts or not under the guidance of experts), this experience is put into different experience pools. It is assumed that expert experiences are generated under the guidance of different human experts. However, different experts may have different UAV motion planning policies. For example, Expert 1 requires the UAV to always maintain a slow speed and fly cautiously, whereas Expert 2 requires UAV to fly at the maximum speed when the threats are not detected, etc. Under the framework of MEP–DDPG, the experiences generated by a UAV interacting with the environment under the guidance of Expert 1 is stored in experience pool 1, the experiences generated under the guidance of Expert 2 is stored in experience pool 2, and so on. The expert experiences generated under the guidance of X−1 different experts are stored in X−1 different experience pools, and the experiences gained by the UAV is stored in experience pool X. During the learning time, a total of N experiences are sampled from X different experience pools to form a batch according to a certain sampling strategy. The parameters of the critic network are updated by minimizing the loss function $L\left(\theta^{Q}\right)$ (Formula (11)). The strategy gradient method is used to update the parameters of the actor network (Formula (12)). Then, the parameters of the target-net in both actor network and critic network are updated by the soft update method. The above process is performed at each time step, and the cycle is repeated until the networks reach convergence.

In the early stages of training, because the UAV knows nothing about the environment, most of the experiences gained by randomly taking actions to interact with the environment is not useful. Therefore, this MEP–DDPG framework uses expert guidance to generate expert experiences and stores them into the corresponding experience pool based on a certain proportion η. As the training process progresses, η gradually decreases to 0 while the learning method gradually returns to the initial DDPG in the second half of the training.

Because the UAV learns higher-quality experiences (expert experiences) in the early stages of training, this means that the parameters of the neural network are quickly adjusted to reach a good state. This method not only avoids falling into a local optimal solution in the early stages of training, but also greatly improves the convergence speed of the neural networks.

### 4.3. MPC-SA for Expert Experiences

During each training process, the UAV interacts with the environment millions of times. If a real expert guides the motion of the UAV to provide expert experiences, it will require a significant amount of manpower and time. Therefore, an MPC-SA framework is proposed, which can simulate human experts by using limited information and provide expert guidance for the UAV’s training.

#### 4.3.1. Model Predictive Control

According to references [44,45,46], the main idea of MPC is rolling optimization. Using the UAV’s AMP as an example and assuming that UAV’s actions are discrete, only three actions can be selected at each moment: deflecting to the left by a certain angle, deflecting to the right by a certain angle, or maintaining the current direction.

**Figure 6:**
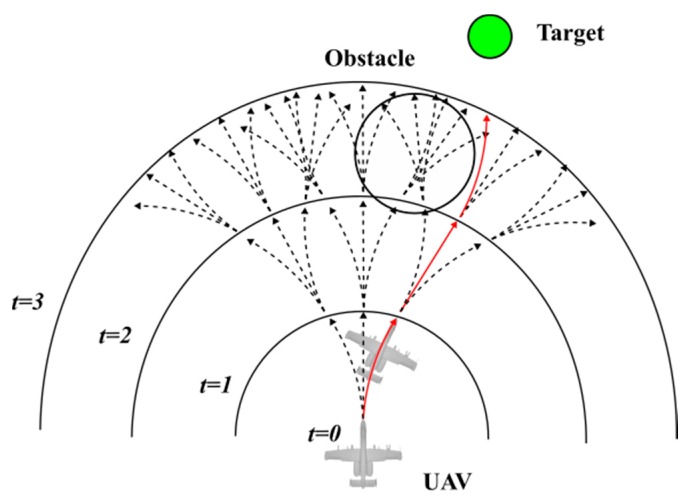
MPC for a UAV’s motion planning (black arrows represent optional action sequences; red arrow represents the optimal action sequence).

As shown in Figure 6, assuming the current time is t=0, all possible action sequences from t=0 to t=n−1 can be enumerated (only the possible action sequences for n=3 are shown). In other words, at each time step, all possible action sequences for the next 3 time steps are enumerated. From these, it is easy to select the optimal action sequence which is shown in red in Figure 6. By executing the red action sequence, the UAV was able to approach the target as quickly as possible without colliding with the obstacle. However, only let the UAV perform the first action in this sequence, which was to deflect to the right by a certain angle at time t=0. Consequently, it reached the position shown at time t=1 in the figure. Then, predict the action sequences of the next 3 time steps and let the UAV execute only the first action again. This process was repeated until the UAV finds the target and completes its mission.

In this way, the MPC rolling optimization method was used to plan the UAV’s motion. This not only helps the UAV select the optimal action at each time step but also avoids failure to complete the task due to the changes in the environment. However, in practice, the action space of the UAV at each time step is continuous, which means that there are countless action sequences that can be selected. It is impossible to list all the action sequences one by one, so it is necessary to use some optimization methods to choose a better action sequence.

#### 4.3.2. Simulated Annealing

Simulated annealing is an optimization algorithm proposed by Kirkpatrick [47] in 1983 that includes two steps. First, a solution is randomly selected to start the process, and then a random perturbation is generated in order to obtain a new solution. Second, the objective function is used to judge whether the new solution is good or bad, and each new solution is selected to be accepted or rejected according to the following Formula (14):

(14) $$p=\{\begin{array}{cc} 1 & if\;E\left(x_{new}\right)<E\left(x_{old}\right)\\ exp\left(-\frac{E\left(x_{new}\right)-E\left(x_{old}\right)}{T}\right) & if\;E\left(x_{new}\right)\geq E\left(x_{old}\right) \end{array}$$

where p is the probability of choosing the new solution to take the place of the old one, while E(x) is the objective function value of the solution x. By simulating the process of object cooling, the process described above is continuously repeated until the cycle ends and the final solution is obtained.

**Figure 7:**
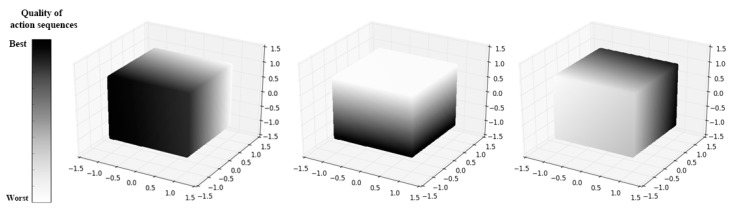
The UAV’s decision-making processes at different states. **Left**: the position of the UAV is (300, 400, 80) and the position of the target is (0, 0, 0); **Middle**: the position of the UAV is (150, 350, 70) and the position of the target is (120, 360, 0); **Right**: the position of the UAV is (0, 0, 20) and the position of the target is (10, 250, 0);.

Because random perturbations are added into the solution process, the SA can move out of the local optimum to determine the global optimal solution. The SA algorithm is used to select among the action sequences predicted at each time step. Figure 7 shows some of the selection processes of the UAV in different states:

Since the dimension of the action space was too high when n≥2 in the MPC, here this paper set n=1 to visualize the decision process which means that the UAV chose the optimal one among all possible action sequences at the current time step and executed it. At each time step, the required load factor of the UAV was a three-dimensional vector whose value range in each direction was [−1,1]. As shown in Figure 7, the action space of the UAV at each moment was a cube, in which there were countless actions that could be selected. Each action sequence was evaluated according to the objective function in the SA algorithm and assigned a color; the darker the color, the better the action sequence. The role of SA was to choose the action sequence with the highest quality (the darkest color) in this continuous action space as much as possible.

### 4.4. MEP–DDPG Algorithm

The experiences generated by the MPC-SA can provide a good approximation for the experiences of human experts. These experiences were placed in different experience pools, and a certain sampling strategy was used for sampling. After applying the DDPG algorithm to train the UAV, the MEP–DDPG algorithm could finally formed (Algorithm 1):
**Algorithm 1: MEP–DDPG Algorithm**Randomly initialize critic network $Q\left(s,a|\theta^{Q}\right)$ and actor $\mu \left(s|\theta^{\mu }\right)$ with weights $\theta^{Q}$ and $\theta^{\mu }$ Initialize target network $Q^{\prime }$ and $\mu^{\prime }$ with weights $\theta^{Q^{\prime }}\leftarrow \theta^{Q}$, $\theta^{\mu^{\prime }}\leftarrow \theta^{\mu }$ Initialize experience pools $R_{1},R_{2}\cdots R_{X}$**for** episode = 1, M
**do** Initialize a random process N for action explorationReceive initial observation state $s_{1}$
**for**t=1,T**do** According to probability η, select action $a_{t}=\mu \left(s_{t}|\theta^{\mu }\right)+N_{t}$ based on current policy and exploration noise or select action $a_{t}$ by MPC-SA Execute action $a_{t}$ and observe reward $r_{t}$ and observe new state $s_{t+1}$
 Store experience $\left(s_{t},a_{t},r_{t},s_{t+1}\right)$ in specific R depending on the source of $a_{t}$. Follow a specific sampling policy, sample a random minibatch of N experiences $\left(s_{i},a_{i},r_{i},s_{i+1}\right)$ from $R_{1},R_{2}\cdots R_{X}$
 Set $$y_{i}=r_{i}+\gamma Q^{\prime }\left(s_{i+1},\mu^{\prime }\left(s_{i+1}|\theta^{\mu^{\prime }}\right)|\theta^{Q^{\prime }}\right)$$
 Update critic by minimizing the loss: $L=\frac{1}{N}\underset{i}{\sum }{\left(y_{i}-Q\left(s_{i},a_{i}|\theta^{Q}\right)\right)}^{2}$
 Update the actor policy using the sampled policy gradient: $\nabla_{\theta^{\mu }}J\approx \frac{1}{N}\underset{i}{\sum }\nabla_{a}Q\left(s,a|\theta^{Q}\right){|}_{s=s_{i},a=\mu \left(s_{i}\right)}\nabla_{\theta^{\mu }}\mu \left(s|\theta^{\mu }\right){|}_{s_{i}}$ Update the target networks: $\theta^{Q^{\prime }}\leftarrow \tau \theta^{Q}+\left(1-\tau \right)\theta^{Q^{\prime }}$
$$\theta^{\mu^{\prime }}\leftarrow \tau \theta^{\mu }+\left(1-\tau \right)\theta^{\mu^{\prime }}$$
 **end for****end for**

At each time step t, the UAV chose an action $a_{t}$ based on the existing strategy or according to the MPC-SA based on a certain probability η. Subsequently, the UAV executed this action $a_{t}$ at the current state of the environment $s_{t}$ and obtained the environmental feedback reward $r_{t}$ and the next state $s_{t+1}$. Next, according to the source of the action $a_{t}$, it stored the experience $\left(s_{t},a_{t},r_{t},s_{t+1}\right)$ in a corresponding experience pool R for the learning process. During the learning time, a total of N experiences were sampled from all experience pools to form a minibatch following a certain policy. Finally, the parameters of each network were updated according to the DDPG’s soft update method.

## 5. Training and Testing Environment

**Figure 8:**
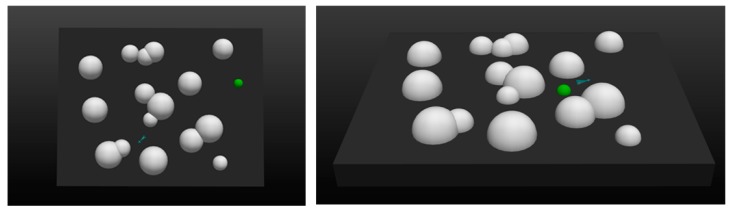
The simulation environment as shown from different perspectives. **Left**: top view; **Right**: main view.

To achieve conditions similar to real-world conditions, this study designed a 3D simulation environment based on the parameters of the real UAV. At present, countries around the world have made progress in the research of UAVs and created advanced UAVs. These advanced UAVs can be used in many different tasks such as: disaster monitoring, anti-smuggling, environmental protection, meteorological observation, forest fire prevention and geological survey, etc. The ranges of some main parameter of these advanced UAVs are shown in the Table 1:

Based on these parameters, a 120 × 90 × 10 km^3^ simulation environment in equal proportions was constructed. The programming language used to build the platform was Python 2, and the display interface was implemented by using the visualization toolkit. Figure 8 shows the simulation environment from different perspectives:

In this environment, the white hemispheres represent the danger areas and the green hemisphere represents the target. The mission of UAV was to fly as low as possible and reach the target area as quickly as possible without entering the danger areas. The UAV’s maximum flight speed was 103 m/s and the maximum load factor was 25. In addition, the acceleration of gravity in the environment was set at a fixed value of 9.8 m/s^2^. The radiuses of the threat areas ranged from 5–10 km while the radius of the target area was 3 km.

**Figure 9:**
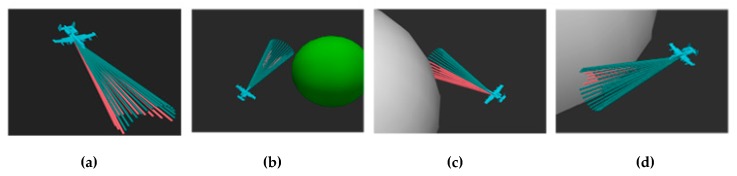
Blue line segments show the sensor range of the UAV. (**a**,**b**): ground (boundary) detected; (**c**,**d**): obstacle detected.

For detection, $N_{r}$ line segments were used to simulate lidar. As shown in Figure 9, $N_{r}$ blue line segments were used to sense the information in environment. Each line segment can calculate the distance d from the UAV to the obstacles or boundaries. In addition, when an object is detected by a line segment, the blue color will change to red as an alert. The monitoring distance of lidar is set to 50 km in the experiments.

At each time step t, an environment state vector $\xi_{e}={\left[d_{1},d_{2},\cdots ,d_{Nr}\right]}^{T}$ was calculated and used to form the state $s_{t}$ together with $\xi_{u}$ and $\xi_{T}$, where $\xi_{T}={\left[p_{Tx},p_{Ty},p_{Tz}\right]}^{T}$, and $p_{Tx}$, $p_{Ty}$, $p_{Tz}$ represent the position of the target. Therefore, the system state $s=\left[p_{Tx}-p_{ux},p_{Ty}-p_{uy},p_{Tz}-p_{uz},\varphi_{u},\alpha_{u},∥v_{u}∥,d_{1},d_{2},\cdots ,d_{Nr}\right]$ with a total of $N_{r}+6$ elements was fed into the critic network, and an action $a_{t}=\left[n_{ux},n_{uy},n_{uz}\right]$ was the output.

To avoid the sparse reward problem in RL, the reward function was set as follows:

(15) $$r\left(s,a\right)=\{\begin{array}{cc} r_{a} & if\;arrive\;the\;target\;area\\ r_{c} & if\;collied\;or\;out\;of\;range\\ \lambda_{1}\left(D_{pre}-D_{cur}\right)+\lambda_{2}\left(-\frac{\Delta \varphi +\Delta \mu }{8}\right)+\lambda_{3}\left(1-\frac{p_{uz}}{H_{env}}\right)+\lambda_{4}d_{ave}+\lambda_{5}∥v_{u}∥ & every\;step \end{array}$$

where $D_{pre}$ and $D_{cur}$ are the previous and current relative distances between UAV and the target, respectively; Δφ and Δμ represent the yaw angle and the pitch angle between UAV’s direction and target, respectively; $H_{env}$ is the maximum height of the simulation environment; $d_{ave}$ represents the average of $\xi_{e}$. These 5 sub items in Formula (15) represent the contributions of distance, angle, height, threat, and velocity to the rewards, respectively, and the contribution rates can be tuned by $\lambda_{1},\lambda_{2}\cdots \lambda_{5}$.

## 6. Experiments and Tests

### 6.1. Training in Static Environments

During the entire training process, the hyper parameters were set as follows:

1. DDPG algorithm:

$N_{r}$ was 32, which indicates that there were 32 line segments used to simulate lidar, and the input layer of the actor network had 38 nodes. The actor network had two hidden layers with 100 nodes for each and one output layer with three nodes, while the critic network had two hidden layers with 100 nodes for each and one output layer with 1 node. The learning rates of the actor and the critic networks were 0.0001 and 0.001, respectively. The soft update rate τ of the actor network was 0.1 and the τ of the critic network was 0.2. The discount factor γ was 0.9. The optimizer was Adam. The size of experience pool was 50,000 and the batch size was 256. The parameters in the reward function (Formula (15)) were: $r_{a\;}=100$, $r_{c}=-200$, $\lambda_{1}=20$, $\lambda_{2}=20$, $\lambda_{3}=10$, $\lambda_{4}=40$, and $\lambda_{5}=10$.

2. MEP–DDPG algorithm:

For the SA part, the objective function was set according to Formula (16):

(16) $$f_{SA}=\rho_{1}\left(D_{pre}-D_{cur}\right)+\rho_{2}D_{obs}$$

where $D_{obs}$ represents the distance between the UAV and the nearest obstacle that can be detected, and the values of $\rho_{1}$ and $\rho_{2}$ are 100 and 200, respectively. The number of extra experience pools for storing expert experiences was 1, and the capacity was 50,000. The sampling policy in multiple experience pools was as follows: k was the proportion of the experiences sampled from the expert experience pools, and its initial value was 100%. During the training process, the value of k decreased by 0.002% at each learning time until it was reduced to 10%. This ensured that at the beginning of the training, a majority of the learning experiences came from the expert experience pools, and only 10% of the experiences came from the expert experience pools in the second half of the training process. All other hyperparameters were the same as those used in the DDPG algorithm.

**Figure 10:**
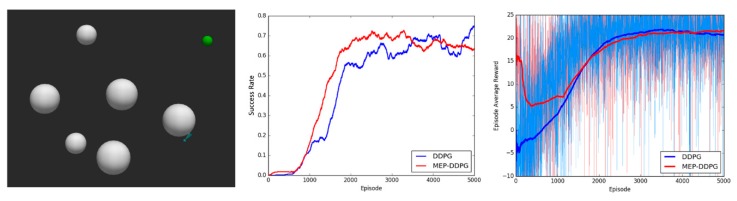
Experimental results in a low complexity environment. **Left**: the simulation environment with low complexity (6 threats); **Middle**: the success rate of DDPG and MEP–DDPG; **Right**: the episode average reward of DDPG and MEP–DDPG.

**Figure 11:**
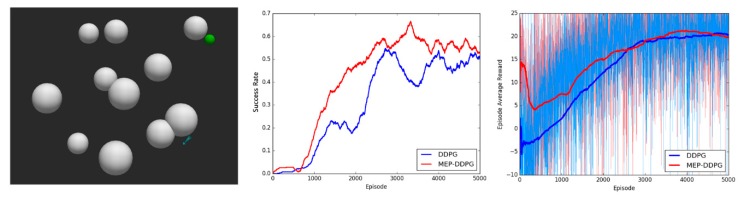
Experimental results in a medium complexity environment. **Left**: the simulation environment with medium complexity (11 threats); **Middle**: the success rate of DDPG and MEP–DDPG; **Right**: the episode average reward of DDPG and MEP–DDPG.

**Figure 12:**
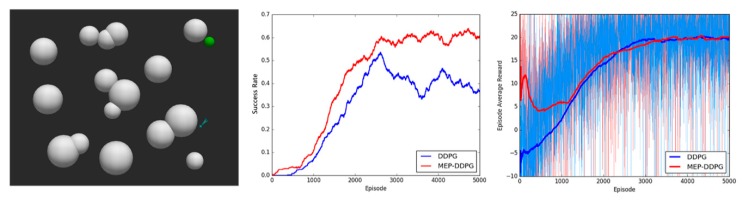
Experimental results in a high complexity environment. **Left**: the simulation environment with high complexity (16 threats); **Middle**: the success rate of DDPG and MEP–DDPG; **Right**: the episode average reward of DDPG and MEP–DDPG.

**Figure 13:**
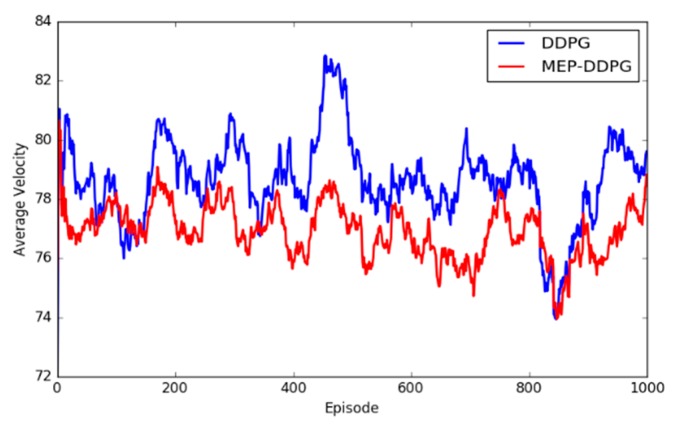
Average velocity of UAVs trained by DPPG and MEP–DDPG.

**Figure 14:**
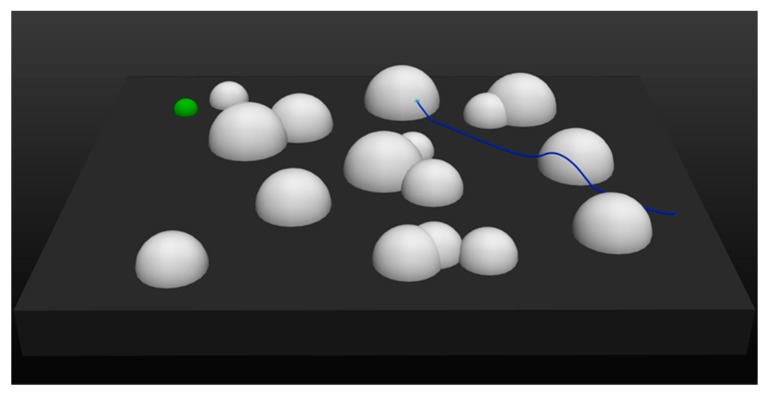
The trajectory of a UAV trained by DDPG.

**Figure 15:**
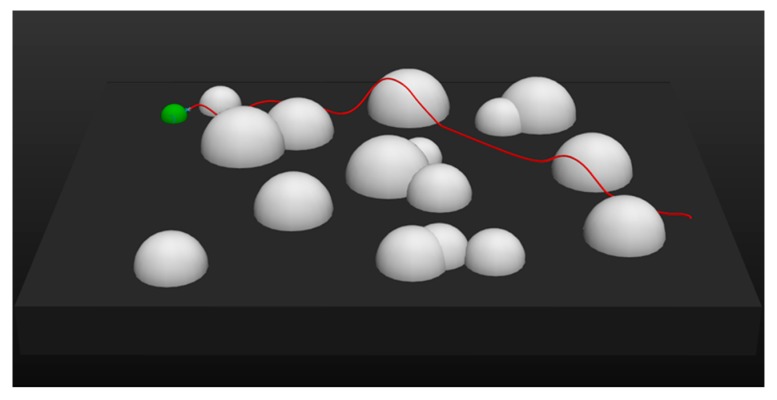
The trajectory of a UAV trained by MEP–DDPG.

During the training process, the position of the UAV and the target were randomly generated. All the algorithms were implemented with Tensorflow and models were trained on Ubuntu 16.04 with a GeForce RTX 2070 GPU for 5000 episodes. The maximum number of steps per episode was set to 3000. It took 10–14 h to complete a training with 5000 episodes. Experiments were performed in simulation environments with different levels of environmental complexity, and the average success rate (the probability of the UAV successfully reaching the target area in last 500 episodes) and episode average reward (the average rewards of all the steps in one episode) were used to evaluate the performance of the algorithms. The experimental results are shown in Figure 10, Figure 11 and Figure 12:

The experimental results demonstrated that the convergence speed and convergence results were better for the MEP–DDPG algorithm than DDPG in different environments. This is because the UAV learns from higher quality experiences (expert experiences) at the beginning of training, which means that the parameters of the neural network are quickly adjusted to an optimal state. However, it is easy to find that the episode average reward of the two algorithms was not significantly different, and sometimes the average reward for the DDPG was higher than that of the MEP–DDPG. This shows that the action selected by the DDPG was no worse than the action selected by the MEP–DDPG in each step, and sometimes it is even better than the MEP–DDPG selected actions. However, at the end of an episode, the UAV trained by DDPG was not able to reach the target position. The models trained by the two algorithms were run 1000 times each, and the average speed of every episode was recorded (Figure 13):

Obviously, the UAV trained by DDPG had a higher velocity than that trained by MEP–DDPG, which leads to the conclusion that in some cases, the UAV trained by DDPG cannot maneuver as safely as the UAV trained by MEP–DDPG when facing the same threats. This is demonstrated in Figure 14 and Figure 15:

It is evident that for the same task, the flight trajectories of UAVs trained by DDPG and MEP–DDPG in the first half are almost identical. However, due to the faster flight speed, the UAV trained by DDPG was unable to slow down and climb in a short period of time when facing threats, which resulted in a failure of the mission. In comparison, the UAV trained by the MEP–DDPG algorithm was able to easily climb to avoid threats and successfully complete tasks. These results demonstrate that DDPG is stuck in a local optimal solution, which leads to the excessively fast flight speed of the UAV. In the real world, flying at a higher velocity is good, because the UAV can complete the task faster; however, the success of the task and the safety of the UAV are more important. Therefore, the more cautious UAV trained by the MEP–DDPG algorithm may have a higher reference value.

### 6.2. Testing for Different Tasks

To test the adaptability of the proposed algorithm, two tasks to simulate what might happen in a real unknown environment were designed.

#### 6.2.1. Testing for Tasks with Sudden Threats

**Figure 16:**
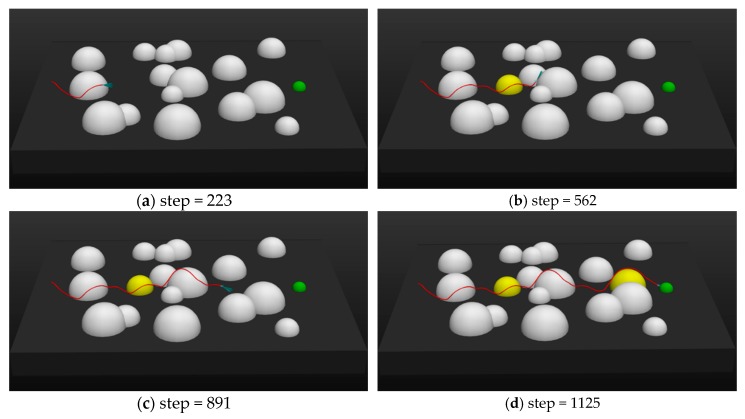
The trajectory of the UAV at different time steps when performing a task with sudden threats.

In a real environment, changes in the dynamic environment can suddenly show up. For example, when a UAV is delivering an item, a car could suddenly pass by. Therefore, carrying out tasks in the presence of sudden threats was simulated:

As shown in Figure 16, the initial positions of the UAV and the target were (500, 10, 30), (−430, −5, 0). After the UAV bypassed the first threat at step = 250, a new threat suddenly appeared at (200, −5, 0) (the yellow hemisphere shown on the left in Figure 16b). When the step increased to 900, another threat suddenly appeared at (−310, 13, 0) (the yellow hemisphere shown on the right in Figure 16d). Judging from the trajectory of the UAV (red lines) in the figure, the UAV was able to make a good judgment and pass through the dangerous area safely when faced with sudden threats.

#### 6.2.2. Testing for Tasks with Moving Target

**Figure 17:**
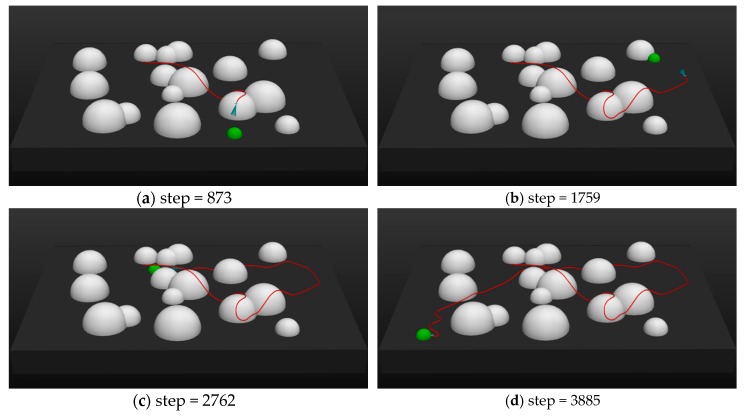
The trajectory of the UAV at different time step when performing task with moving target.

This test was used to simulate the moving target problem in UAV’s AMP. In many application scenarios, the target area to be reached can suddenly changes. Changes of the target area may cause the failure of the UAV’s task. The following test can simulate this situation. Figure 17 presents the testing process:

As shown in the figures above, the initial positions of the UAV and the target were (200, 200, 30), (−170, −410, 0). In Figure 17a, at step = 873, the UAV was very close to the target, but then the target position suddenly changed to (−450, 300, 0) when step = 900. The UAV quickly turned to adjust the attitude and fly to the new target position (Figure 17b). When step = 1800, the position of target changed again to (150, 200, 0) (Figure 17c). When step = 2700, the position of the target changed for the third time to (500, −400, 0) (Figure 17d). The UAV’s entire flight trajectory (red lines) demonstrated that, even though the position of the target changed several times, the UAV was still able to reach the target position safely and accurately. Therefore, the moving target problem was addressed successfully.

## 7. Conclusions and Future Work

This paper proposed a MEP–DDPG algorithm to address UAV’s AMP problem. By using an MPC-SA framework to generate expert experiences and multiple experience pools to store expert experiences, MEP–DDPG can go beyond the local optimum generated during the early stages of training. Experimental results show that the convergence speed and results of the MEP–DDPG algorithm are better than those of the DDPG algorithm in several different complex unknown environments. In the testing process, MEP–DDPG performed well in some common tasks that may occur in real situations. 

In the future, we intend to add additional complex tasks such as multi-target tracking to our 3D simulation environment. In addition, we plan to make the location and scope of the threat change over time to more realistically simulate some application environments.

## Figures and Tables

**Table 1:** The ranges of some main parameter of some advanced UAVs.

Parameter	Range
maximum flying height	7000–12,000 m
maximum flight speed	220–370 km/h
maximum flight time	20–40 h
maximum load factor	20–30
maximum take-off weight	3–7 t
